# Untargeted Metabolomics Revealed Potential Biomarkers of Small Yellow Follicles of Chickens during Sexual Maturation

**DOI:** 10.3390/metabo13020176

**Published:** 2023-01-26

**Authors:** Jinbo Zhao, Hongbin Pan, Wenjiang Zhao, Wei Li, Haojie Li, Zhongxiao Tian, Dayong Meng, Yuting Teng, Xinlu Li, Yang He, Hongmei Shi, Changrong Ge, Kun Wang

**Affiliations:** 1 Faculty of Animal Science and Technology, Yunnan Agricultural University, Kunming 650201, China; 2 Branch of Animal Husbandry and Veterinary of Heilongjiang Academy of Agricultural Sciences, Qiqihar 161005, China

**Keywords:** untargeted metabolomics, sexual maturation, small yellow follicles, ferroptosis, mTOR signaling pathway, differentially expressed metabolites and metabolic pathways, potential biomarkers

## Abstract

Sexual maturation provides economically important traits in poultry production. Research on the initiation mechanism of sexual maturity is of great significance for breeding high-yield laying hens. However, the underlying mechanisms are not fully clear. Here, one hundred and fifty Chahua No. 2 laying hens (the CH2 group, which has precocious puberty) and one hundred and fifty Wu Liang Shan black-bone laying hens (the WLS group, a late-maturing chicken breed) with similar weights and ages were randomly selected. ELISA was used to determine the secretion levels of luteinizing hormone (LH), estradiol (E2), and progesterone (P4) in 150-day-old serum and small yellow follicle (SYF) tissues. A histology examination, immunohistochemistry, and quantitative real-time PCR (qPCR) were used to explore the molecular mechanism of how some genes related to oxidative stress affect sexual maturation. The results showed that the secretion levels of LH, E2, and P4 in the CH2 group serum and SYF were higher than those in the WLS group. The results of the real-time PCR of all genes showed that the expression levels of cytochrome P450 family 11 subfamily A member 1, steroidogenic acute regulatory protein, follicle-stimulating hormone receptor, and cytochrome P450 family 19 subfamily A member 1 in the CH2 group were significantly higher than those in the WLS groups (*p* < 0.001). Untargeted metabolomics combined with multivariate statistical analysis was used to identify biomarkers of SYF tissues in the CH2 and WLS groups. A trajectory analysis of the principal component analysis (PCA) results showed that the samples within the group were clustered and that the samples were dispersed between the CH2 and the WLS groups, indicating that the results of the measured data were reliable and could be used for further research. Further analysis showed that a total of 319 metabolites in small yellow follicles of the CH2 and WLS groups were identified, among which 54 downregulated differential metabolites were identified. These 54 metabolites were found as potential CH2 biomarkers compared with WLS at 150 days, and the different expressions of L-arginine, L-prolinamide, (R)-4-hydroxymandelate, glutathione, and homovanillic acid were more significant. Twenty metabolic pathways were found when significantly differential metabolites were queried in the KEGG database. According to the impact values of the metabolic pathways, eighteen differential metabolites belonged to the mTOR signaling pathway, glutathione metabolism, ABC transporters, the cell ferroptosis pathway, and D-arginine and D-ornithine metabolism. Interestingly, we identified that the cell ferroptosis pathway played an important role in chicken follicle selection for the first time. The histology and immunohistochemistry of SYF showed that the number of granulosa cells increased in the CH2 groups and the expression levels of glutathione peroxidase 4, tumor protein p53, ribosomal protein S6 kinase, and sterol regulatory element binding protein 1 in the granulosa cell layer were upregulated in the CH2 group at the time of sexual maturation. Furthermore, we also speculated that the antioxidant system may play an indispensable role in regulating sexual maturity in chickens. Overall, our findings suggest differentially expressed metabolites and metabolic pathways between CH2 and WLS chickens, providing new insights into the initiation mechanism of sexual maturation.

## 1. Introduction

Sexual maturation is an economically important trait for poultry genetics and breeding that is not only related to genetic factors but is also closely related to the metabolic status of the body [1]. Furthermore, sexual maturation is related to follicle development and differentiation in the ovaries [2]. The ovaries of chickens begin to grow at approximately 14 weeks old and attain sexual maturity between the ages of 18 and 20 weeks [3,4]. When poultry reaches sexual maturity, a dominant follicle is selected from the pool of pre-hierarchical follicles to complete the physiological process of laying eggs every day [5]. During hens’ reproductive lives, most ovarian follicles undergo atresia, with only approximately 5% progressing to the final hierarchical stages of maturation and ovulation [6]. The function of the ovaries of hens is regulated by the “hypothalamus-pituitary-ovarian” axis. Both the granular layer and the thecal layer of the ovary are regulated by gonadotropins, such as FSH and LH, which are produced in the pituitary gland. Among them, LH is responsible for the maturation of pre-hierarchical follicles [7], and FSH is responsible for ovarian growth in the initial stage of development [8].

In particular, small yellow follicle development is a key process for improving the reproductive performance of chickens [9]. Small yellow follicles are composed of a granulosa cell layer, a thecal cell layer, and an oocyte; steroid hormone synthesis is considered to be a three-cell model; FSH-mediated cAMP signaling can promote differentiated GCs; *StAR* mRNA expression and StAR can synthesize progesterone in coordination with luteinizing hormone (LH) in the hierarchical follicle (the largest ovarian follicle) [10]; testosterone hormones are secreted by the inner layer of thecal cells; and estrogen hormones are secreted by the thecal cells of the second and third largest ovarian follicles [11]. These processes are regulated by several hormones [12,13]. It is widely believed that the expression levels of *FSHR* and *cAMP* in granulosa cells are important biological markers for follicle selection in laying hens [14], and the expression level of *FSHR* mRNA is the highest in 6–8 mm small yellow follicles compared to immature follicles [15]. Sexual maturation and ovary development are not only regulated by several growth factors, such as growth differentiation factor 9 [16], Wilms tumor gene 1 [17], gremlin 1 gene [18], and forkhead box L2 [19]. They are also regulated by insulin-like growth factor [20], epidermal growth factor [21], and vasoactive intestinal peptide [22]. Research has shown that miRNAs such as *miR-25b-3p* [23], *miR-449b-5p* [24], and *miR-135a-5p* [25] play an important role in sexual maturation and ovary development. At present, studies have mainly focused on transcriptome analyses of differentially expressed genes of follicle selection in domestic chickens.

Due to its high resolution and sensitivity [26], untargeted metabolomics has been widely adopted in the fields of disease diagnosis, biomedicine, and toxicology [27,28]. Metabolomics was considered to be the bridge between genomics and phenotypes and was the most direct manifestation of explaining complex biological traits [29]. However, differentially expressed metabolites and enrichment metabolic pathways in the field of poultry reproduction have been less clearly understood. 

In this study, we wanted to identify the biomarkers of small yellow follicles in different breeds, namely a new breed with precocious puberty (CH2) and a late-maturing local breed (WLS), to obtain differentially expressed metabolites and metabolic pathways using untargeted metabolomics. Moreover, a histology examination, immunohistochemistry, and quantitative real-time PCR (qPCR) were used to explore the molecular mechanisms of some genes related to sexual maturation in the CH2 and the WLS chickens. This study was intended to provide a new perspective for the breeding of precocious puberty in Cha Hua No. 2 chickens.

## 2. Materials and Methods

### 2.1. Ethics Statements

The present study was conducted in accordance with the recommendations in the guide for the care and use of laboratory animals of the National Institutes of Health, and the protocol was approved by the institutional animal care and use committee (approval ID: YAUACUC01, publication date: 10 July 2013) at Yunnan Agriculture University.

### 2.2. Animals and Experimental Design

A total of 300 laying hens (Cha Hua No. 2 chickens, *n* = 150; Wu Liang Shan chickens, *n* = 150) were obtained at the age of 150 days from the Yunnan Agricultural University chicken farm. The Cha Hua No. 2 chickens were referred to as the CH2 group, and the Wu Liang Shan chickens were referred to as the WLS group. All chicks were maintained in individual cages (450 mm × 400 mm × 380 mm). The temperature was maintained at 23 °C ± 2 for laying hens, and the average relative humidity was 50%. Laying hens were given ad libitum access to clean and fresh water and a corn–soybean meal basal diet. The dietary composition and nutrient levels can be seen in Table 1.

### 2.3. Sample Collection and Preparation

At the age of 150 days, 10 hens were randomly selected from the CH2 and WLS groups. Blood samples were collected from the wing veins of CH2 and WLS chickens, and they were euthanized by cervical dislocation. The blood samples were centrifuged at 3000 r/min for 15 min to separate serum and were frozen at −20 °C to analyze the levels of LH, E2, and P4 in the serum. LH, P4, and E2 were measured with commercial ELISA kits (Jiangsu Mei mian industrial Co., Ltd., Taizhou, China) in accordance with the manufacturer’s instructions. Ovaries were immediately removed, and small yellow follicles were separated from different pre-hierarchical follicles and were frozen at −80 °C for the determination of LH, E2, and P4 in small yellow follicles, tissue secretion levels, and untargeted metabolomics. 

### 2.4. Liquid Chromatography–Mass Spectrometry (LC-MS)-Based Differential Metabolites and Metabolic Pathway Analysis

This study used modern analytical methods such as LC-MS for the identification of metabolites, and the operation was introduced as follows: A liquid chromatography analysis was carried out using a Vanquish UHPLC System (Thermo Fisher Scientific, Waltham, MA, USA). Chromatography was carried out using an ACQUITY UPLC ^®^ HSS T3 (150 × 2.1 mm, 1.8 µm) (Waters, Milford, MA, USA). The parameters were as follows: a flow rate of 0.25 mL/min and an injection volume of 2 μL. For the LC-ESI (+)-MS analysis, the mobile phase consisted of a 0.1% formic acid–acetonitrile solution (C) and a 0.1% formic acid–water solution (D). The gradient elution procedure was as follows: 0–1 min, 2% C; 1–9 min, 2–50% C; 9–2 min, 50–98% C; 12–13.5 min, 98% C; 13.5–14 min, 98–2% C; and 14–20 min, 2% C. For the LC-ESI(-)-MS analysis, the mobile phase was composed of acetonitrile (A) and a 5 mM ammonium formate–water solution (B). The gradient elution procedure was as follows: 0–1 min, 2%A; 1–9 min, 2–50% A; 9–12 min, 50–98% A; 12–13.5 min, 98% A; 13.5–14 min, 98–2% A; and 14–17 min, 2% A [30].

The mass spectrometric detection of metabolites was performed using an Orbitrap Exploris 120 (Thermo Fisher Scientific, Waltham, MA, USA) with an ESI ion source. Positive and negative ion modes were used to collect data. The parameters were as follows: sheath gas pressure, 30 arb; aux gas flow, 10 arb; spray voltage, 3.50 kV and −2.50 kV for ESI (+) and ESI (-), respectively; capillary temperature, 325 °C; MS1 range, *m*/*z* 100–1000; MS1 resolving power, 60000 FWHM; number of data-dependent scans per cycle, 4; MS/MS resolving power, 15000 FWHM; normalized collision energy, 30%; and dynamic exclusion time, automatic [31]. Metabolites were identified by accurate mass (<30 ppm) and MS/MS data, which were matched with data from HMDB (http://www.hmdb.ca) [32], massbank (http://www.massbank.jp/) (accessed on 9 September 2022) [33], LipidMaps [34] (http://www.lipidmaps.org) (accessed on 10 September 2022), mzcloud (https://www.mzcloud.org) (accessed on 10 October 2022) [35], and KEGG (http://www.genome.jp/kegg/) (accessed on 10 November 2022) [36]. R language ropes package (Ropls) [37] software was used for a principal component analysis (PCA). *p* values < 0.05 and VIP values > 1 were considered to be statistically significant. Differential metabolites were subjected to a pathway analysis using Metabo Analyst [38]. The metabolites identified using metabolomics were then mapped to the KEGG pathway. The metabolites and corresponding pathways were visualized using the KEGG Mapper tool.

### 2.5. RNA Extraction and Quantitative Real-Time PCR (qPCR) Analysis

qPCR is a reliable way to study genes [39,40]. In our experiment, qPCR was used to detect the *StAR, CYP11A1, CYP19A1, FSHR, S6K, SCL7A11, GPX4,* and *TP53* genes. Total RNA was extracted from the small yellow follicle tissues of the CH2 and WLS groups. The concentration and purity of total RNA were detected using NanoDrop2000. cDNA was synthesized according to manufacturer’s instructions (Wu han servicebio technology CO., Ltd, China). The mRNA expression levels of the nine abovementioned genes in small yellow follicles in the CH2 and WLS groups were measured using qPCR. The primer sequence synthesis for qPCR was in accordance with the manufacturer’s instructions, as shown in Table 2. All reactions were performed in triplicate, and the relative mRNA abundance was calculated using the 2−*^ΔΔCt^* method [39], with β-actin as the internal reference gene.

### 2.6. Histology Examination and Immunohistochemistry Analysis

SYF tissue blocks were fixed in 4% paraformaldehyde, dehydrated, embedded in wax, and then cut into 5–6 mm thick sections. The sections were stained with hematoxylin-eosin (H&E). Scanning software was applied to acquire full-field high-resolution digital images of the sections. The images were observed using Case Viewer software. For immunohistochemistry, the sections were put into a 3% hydrogen peroxide solution and incubated at room temperature and away from light for 25 min. The slides were placed in PBS (pH 7.4) and washed three times on a decolorizing shakable bed (5 min each time). The tissue was uniformly covered with 3% BSA in the chemical ring and closed at room temperature for 30 min. The sealing solution was gently shaken off, PBS and a primary antibody were added in a certain proportion to the sections, and the sections were placed flat in a wet box at 4 °C for overnight incubation. The slides were placed in PBS (pH 7.4) and washed 3 times on a decolorizing shaker (5 min each time). After the sections were slightly dried, the secondary antibody of the species corresponding to the primary antibody (HRP labeling) was added to cover the tissue in the ring and incubated at room temperature for 50 min. The sections were placed in PBS (pH 7.4) and washed 3 times on a decolorizing shaker (5 min each time). After the sections were slightly dried, a freshly prepared DAB color-developing solution was added to the circle. The color-developing time was controlled under the microscope. The positive color was brown and yellow, and the sections were washed with tap water to terminate the color development. Hematoxylin was redyed for about 3 min and washed with tap water, and the hematoxylin differentiation solution was differentiated for several seconds and rinsed with tap water. The hematoxylin reverting blue solution reverted to blue and was rinsed with running water. All of the sections were rehydrated through a graded ethanol series and underwent antigen repair, and the results were interpreted under a white light microscope. The main basis for the interpretation of the results was that hematoxylin-stained nuclei were blue and DAB-positive expression was brownish yellow. The details are shown in Table 3.

### 2.7. Statistical Analysis

The data obtained in our experiment were analyzed using the SPSS 19.0 (IBM Corporation, Armonk, NY, USA). All data were expressed as means ± standard deviations (SD). Bars with * and ** represent *p <* 0.05 and *p <* 0.01 significance levels, respectively. 

## 3. Results

### 3.1. The Determination of LH, P4, and E2 Secretion Levels in Serum and Small Yellow Follicle Tissue Using Enzyme-Linked Immunosorbent Assay (ELISA)

**Figure 1:**
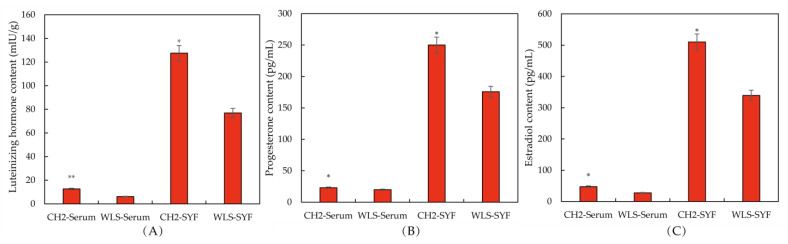
The levels of reproductive hormones in the CH2 and WLS chickens in serum and SYF tissue. (**A**) LH hormone level, (**B**) P4 hormone level, (**C**) E2 hormone level. LH: luteinizing hormone, P4: progesterone, E2: estradiol. * *p <* 0.05, indicates significance compared to the WLS group. ** *p <* 0.01, indicates extreme significance compared to the WLS group.

The levels of reproductive hormone secretion in serum and SYF were determined using ELISA. The purpose of this experiment was to explore whether the sexual maturity of CH2 was earlier than WLS. As can be seen in the Figure 1A, an extremely significant upregulation of LH and E2 levels (*p* < 0.01) in the CH2 group was found compared with the WLS group. A significant upregulation of P4 was found in the CH2 group compared with the WLS group (*p* < 0.05). As shown in Figure 1B,C, on the aspect of SYF, interestingly, there were also significant differences between the CH2 group and the WLS group in the detection of LH, P4, and E2 hormone secretion levels (*p* < 0.05). the levels of LH, E2, and P4 were significantly higher in the CH2 group than in the WLS group.

### 3.2. Expression Levels of Genes Associated with Sexual Maturation in Chicken SYF Tissue

**Figure 2:**
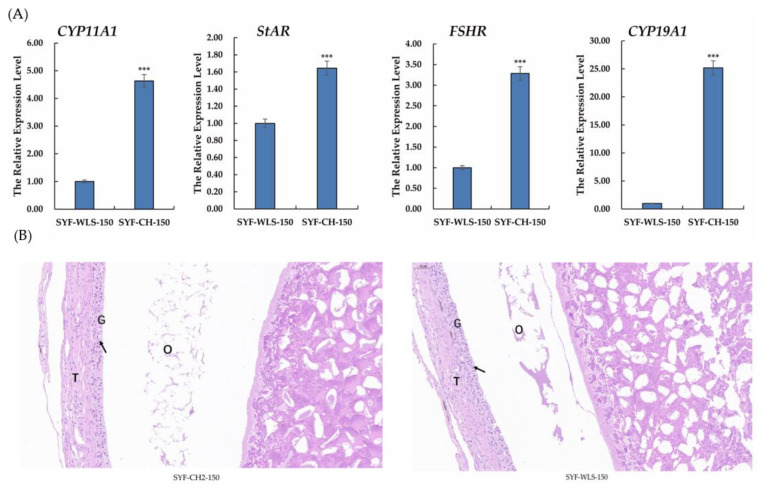
(**A**) Relative mRNA expression levels of genes associated with sexual maturation in SYF tissue. SYF-WLS-150 represents small yellow follicles of Wu Liang Shan at the age of 150 days. SYF-CH-150 represents small yellow follicles of Cha Hua No. 2. at the age of 150 days. *** *p* < 0.001 (**B**) The results of H&E staining, showing the three main parts of SYF. T: thecal cell layer; G: granulosa cell layer; O: oocytes. The figures were imaged at 20× magnification. Arrows point to the granulosa cell layer.

To investigate the expression of genes associated with sexual maturation in chicken SYF tissue, qPCR and histology observations were performed on chicken SYF tissue. As shown in Figure 2A, in our experiment, the results of real-time PCR of all genes showed that the *CYP11A1, StAR, FSHR,* and *CYP19A1* expression levels in the CH2 group were extremely significantly higher than those in the WLS group (*p* < 0.001). Among them, the *CYP19A1* expression level was the highest in the CH2 group. Our data suggest that these genes are involved in the development of sexual maturation, such as *CYP11A1, StAR,* and *CYP19A1,* which is a key enzyme gene associated with steroid hormone synthesis. Follicle-stimulating hormone (FSH) plays an important physiological role in the reproductive performance of chickens by binding to its receptors, which control follicular development and recruitment in the ovary. The results of the experiment showed clear support for the important discovery of precocious puberty in Cha Hua No. 2 chickens. The H&E staining results showed that the SYF of the CH2 group exhibited regular GC and TC morphology, intact structure, and uniform distribution compared to the SYF of the WLS group (Figure 2B). These results suggest that genes associated with steroid hormone synthesis are involved in Cha Hua No. 2 at the time of follicle selection and are the morphological and molecular basis of early sexual maturation in Cha Hua No. 2.

### 3.3. Identification of Potential Differential Metabolites in SYF Tissue

**Figure 3:**
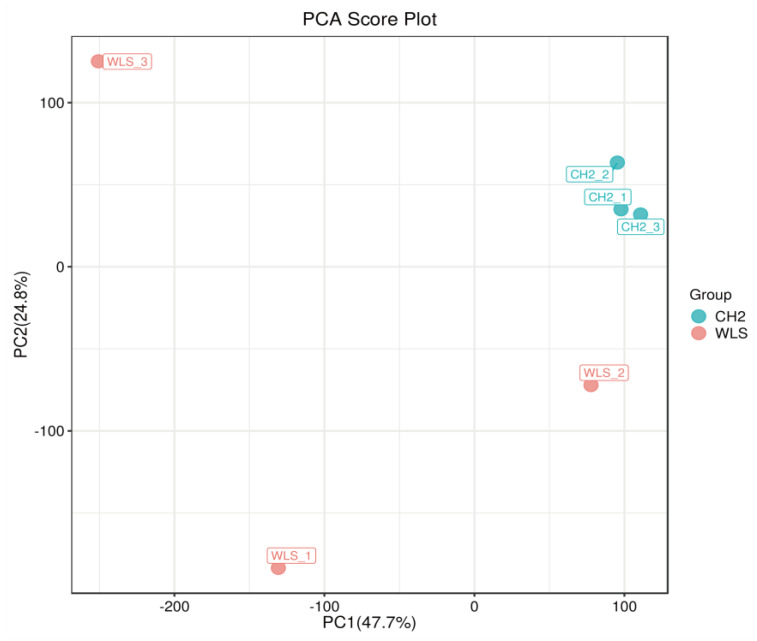
Principal component analysis (PCA) score plot. PC1: the first principal component epla−nation; PC2: the second principal component explanation. The dots represent the samples. Red dots represent the WLS group, and blue dots represent the CH2 group.

PCA was used to reveal changes in the metabolic profiles of samples under different conditions [41]. As shown in the Figure 3, the PCA score plot showed clear separation between the CH2 and WLS groups. Scattered points corresponding to the samples of the CH2 and WLS groups showed mutual aggregation within each group, indicating a good degree of differentiation between the two groups and good repeatability within each group. The above findings indicated that the samples we selected could be used to explore differentially expressed metabolites based on the results of statistical tests (*p* < 0.05 and VIP score > 1.0).

**Figure 4:**
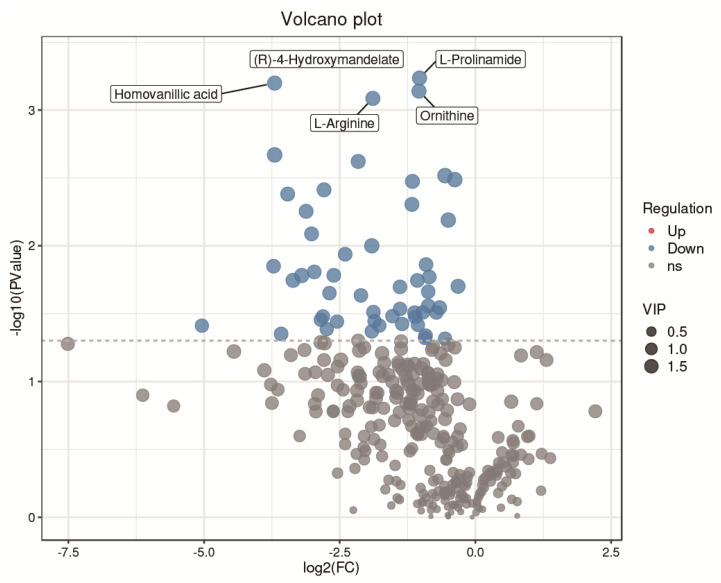
Volcano plot showing the distribution of differential metabolites. Blue represents dow−regulated differential metabolites. Gray represents metabolites that did not meet the filtering parameters. At the top, the names of the five metabolites with the smallest *p* values are displayed.

**Figure 5:**
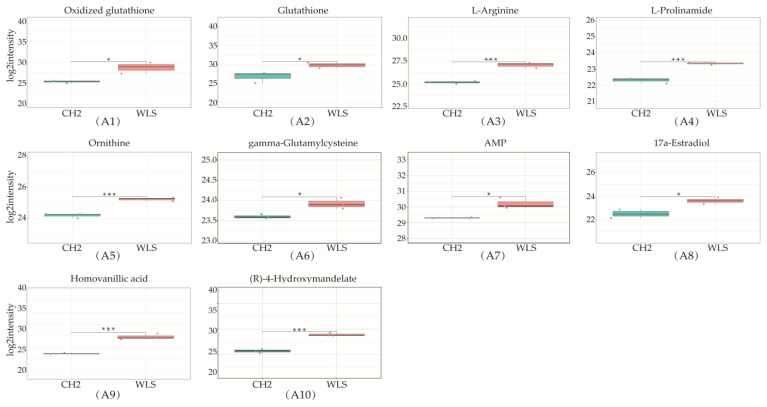

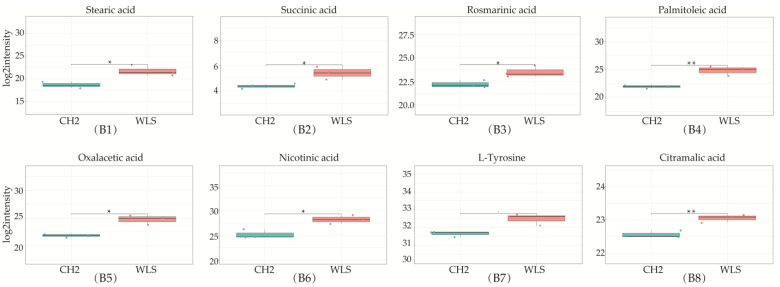
Differentially expressed metabolites in SYF in CH2 and WLS at 150 days. (**A1**–**A10**) Ten differential metabolites were identified (*p* < 0.001 and *p* < 0.05); (**B1**–**B8**) Identified partial differential metabolites that may be associated with sexual maturity, * *p <* 0.05, indicates significance compared to the WLS group. ** *p <* 0.01, indicates extreme significance compared to the WLS group. (*p* < 0.05 and *p* < 0.01). *** *p* < 0.001, indicating extreme significance compared to the WLS group.

A total of 319 metabolites were identified using an untargeted metabolomics technique. It is worth noting that we obtained fifty-four significantly downregulated differential metabolites in the CH2 group in comparison with the WLS group. As can be seen in Figure 4, the differentially expressed metabolite distribution can be visually seen in the volcano plot, in which five differentially expressed metabolites were the lowest (*p <* 0.001), namely L-arginine, L-prolinamide, (R)-4-hydroxymandelate, homovanillic acid, and glutathione. Five differential metabolites decreased in the CH2 group but increased in the WLS group. L-prolinamide and L-Arginine can be synthesized by animal cells and were classified as nutritionally nonessential amino acids (NEAAs), and they reduced the level of ROS and improved the level of glutathione (GSH) in oocytes. This was an important finding in the understanding of Cha Hua No. 2, which was lower in SYF compared with a local late-maturing breed. For the first time, our above findings implied that relatively low amino acid levels may be a characteristic of CH2 chickens, which needs to be further explored in the future. Homovanillic acid is associated with tyrosine metabolism. From the results, we found that Tyr and melanin were higher in the WLS group than in the CH2 group, and Tyr can synthesize melanin, further demonstrating the reliability of our data. (R)-4-Hydroxymandelate is an intermediate involved in fatty acid metabolism (Figure 5). It is worth noting that fifty-four metabolites were identified as potential metabolic biomarkers related to sexual maturation. The differential abundances of these metabolites indicated specific and distinct metabolic profiles between the CH2 and WLS groups at the time of sexual maturation.

### 3.4. Differential Metabolic Pathway Analysis in SYF Tissue

**Figure 6:**
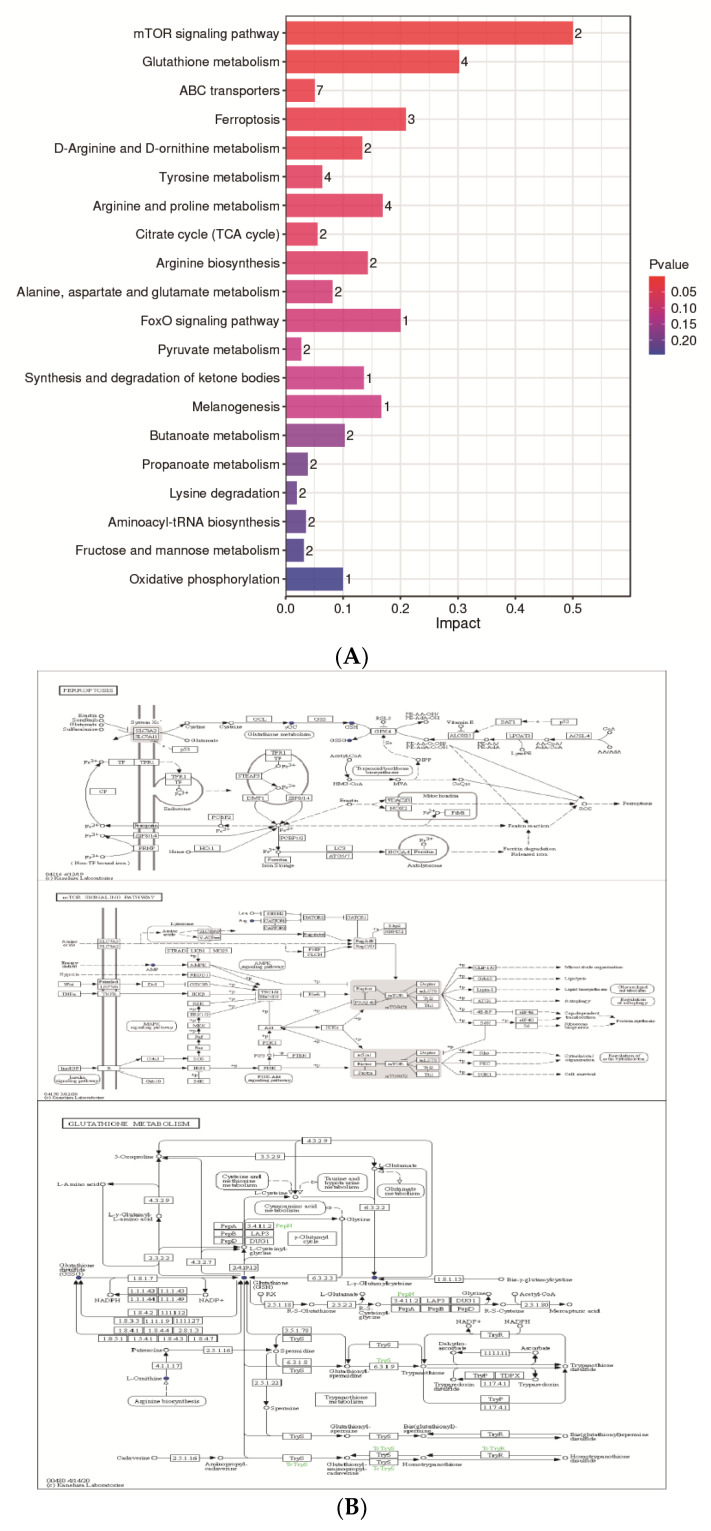
(**A**) KEGG analysis histogram of differentially expressed metabolites. Twenty metabolic pathways were identified based on the *p*-values. Impact values represent the contributions of metabolites detected for these pathways. The higher the impact value, the higher the contribution rate of the metabolites enriched in the pathway. (**B**) The results of untargeted metabolomics on ferroptosis, the mTOR signaling pathway, and glutathione between the CH2 group and the WLS group. Boxes represent protein molecules, and circles represent metabolic molecules. Red indicates the substance was upregulated, and blue indicates the substance was downregulated.

The KEGG database was used to analyze differential metabolites and obtain metabolism-related pathways between CH2 chickens and WLS chickens. Our results, as shown in Figure 6A, indicate that five pathways (the mTOR signaling pathway, glutathione metabolism, ABC transporters, ferroptosis, and D-Arginine and D-ornithine metabolism) were found between the CH2 and WLS groups when significantly differential metabolites were queried in the KEGG database. Eighteen differentially expressed metabolites were enriched in five metabolic pathways. Interestingly, it is apparent in Figure 6B that these important metabolic pathways are related to amino acid transport and metabolism between CH2 and WLS chicken SYF. The most interesting aspect of this figure is that SLC7A11 is not only a key regulator of ferroptosis and the mTOR signaling pathway, but it also maintains cell redox status homeostasis. GSH is a necessary cofactor of GPX4, which is also an important regulatory target for acid metabolism. These two regulators are associated with ferroptosis, the mTOR signaling pathways, and the GSH metabolic signaling pathways (Figure 6B). In addition, we also screened out potential metabolic pathways and differential metabolites related to sexual maturity. As shown in Table 4, AMP and L-arginine were enriched in the mTOR signaling pathway; glutathione, ornithine, and oxidized glutathione were enriched in glutathione metabolism; glutathione, ornithine, oxidized glutathione, and γ-glutamycysteine were enriched in ferroptosis; and glutathione, ornithine, L-arginine, mannitol, sorbitol, 4-hydroxyproline, and methyl-beta-D-galactosidase were enriched in ABC transporters. L-Arginine and ornithine were enriched in D-arginine and D-ornithine metabolism.

### 3.5. Expression Levels of Genes and Proteins Associated with Oxidative Stress in Chicken SYF Tissue

**Figure 7:**
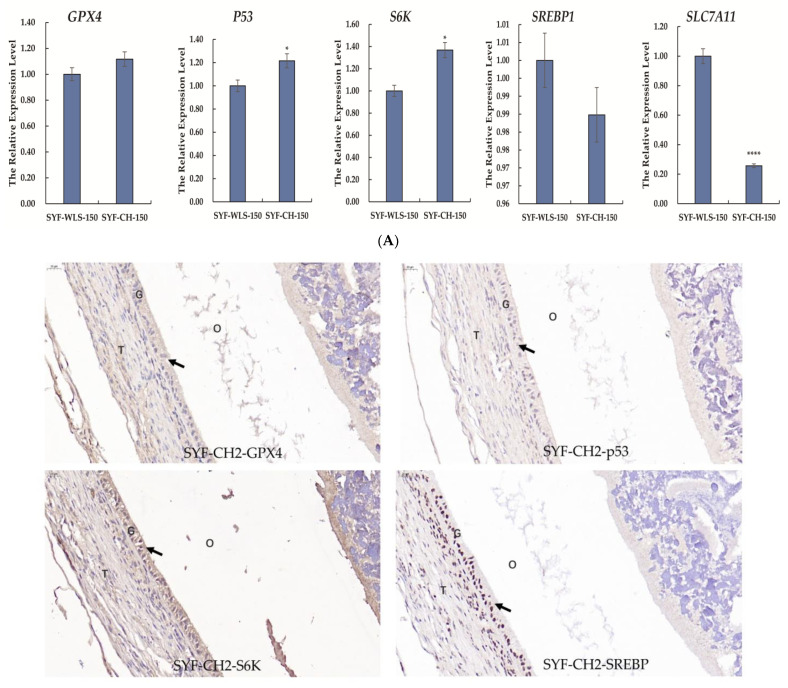

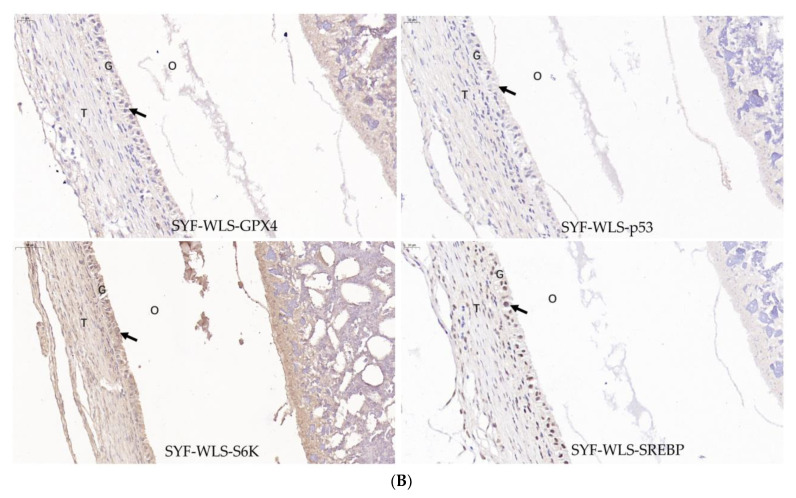
(**A**) Expression levels of genes associated with oxidative stress in chicken SYF tissue. SYF-WLS-150 represents small yellow follicles of Wu Liang Shan at 150 days. SYF-CH-150 represents small yellow follicles of Cha Hua No. 2. at 150 days. * *p* < 0.05, indicating significance compared to the WLS group. **** *p* <0.001 indicating extreme significance compared to the WLS group. (**B**) Expression pattern of protein associated with oxidative stress in chicken SYF tissue (400× magnification) expressed in the granulosa cell layer of SYF. Arrows point to the GC layer of SYF.

Many differential metabolites and metabolic pathways were found using untargeted metabolomics and were related to oxidative stress. Hence, we speculate that oxidative stress plays a vital role in the development of sexual maturation in chickens. We detected mTOR-signaling-related genes (***SREBP1*** and ***S6K***). Ferroptosis-signaling-related genes (***GPX4, p53,*** and ***SLC7A11***) were detected in SYF tissue using quantitative real-time PCR technology. As shown in Figure 7A, interestingly, we found that the expression level of ***SLC7A11*** in the CH2 group was significantly lower compared to the WLS group (***p*** < 0.001). The expression levels of the ***p53*** and ***S6K*** genes in the CH2 group were significantly higher than those in the WLS group (***p*** < 0.05). The expression levels of the ***GPX4*** and ***SREBP1*** genes had no significant differences between the CH2 and the WLS groups (***p*** > 0.05). It was believed that the “***SLC7A11-GSH-GPX4***” axis was the primary cellular system for defense against ferroptosis. In our experiment, the expression of the ***p53*** gene in the CH2 group was higher than in the WLS group. ***p53*** inhibits cystine uptake and sensitizes cells to ferroptosis by repressing the expression of ***SLC7A11***. Similar to the mRNA expression pattern, the ***GPX4***, ***p53***, ***S6K***, and ***SREBP1*** proteins were predominantly expressed in the granulosa cell layer of SYF tissues, according to the immunohistochemistry results (Figure 7B). These results suggest that the protein expression levels of ***GPX4***, ***p53***, ***S6K***, and ***SREBP1*** in the granulosa cell layer were upregulated in the CH2 group at the time of sexual maturation. Hence, from the perspective of the expression levels of genes and proteins, our data indicate that the oxidative stress states of Cha Hua No. 2. chickens can be dynamically regulated by the “***SLC7A11-GSH-GPX4***” axis and can relieve stress from the environment and nutrition so as to maintain the homeostasis of the chicken.

## 4. Discussion

Sexual maturation is fundamental to reproductive and production performance [42] and has been widely used in poultry breeding. Age at first egg (AFE) is used as a phenotype parameter to measure sexual maturity [43]. However, untargeted metabolomics techniques have rarely been used directly in the field of poultry reproduction. Therefore, we used an untargeted metabolomics technique in our experiment to identify and analyze potential biomarkers of SYF for the first time. The results of this experiment proved that fifty-four downregulated differential metabolites may be considered as potential biomarkers of sexual maturation. In our study, we mainly focused on the ferroptosis pathway, the mTOR signaling pathway, and glutathione metabolism.

Reproductive hormone levels were considered to help measure the stage of sexual maturation [44]. In our study, we found that the secretion levels of LH, P4, and E2 in the CH2 group were significantly higher than those in the WLS group in the serum and SYF tissue. Previous research reported that follicle selection is indispensable in the reproductive process in female chickens, and the symbol of this is the proliferation and differentiation of granulosa cells (GCs). In follicular GCs of chickens, the expression of steroidogenic acute regulatory protein *(StAR)* and cytochrome P450 family 11 subfamily A member 1 *(CYP11A1)* is a prerequisite for progesterone synthesis and is related to follicle selection [45,46,47]. LH levels increase significantly after sexual maturity, reaching a peak 30–40 days after egg laying begins. LH, E2, and P4 in plasma work together to complete follicle selection after sexual maturity [48,49]. This result agreed well with previous studies measuring LH, P4, and E2 secretion levels in laying hens. Our findings suggest that CH2 has the characteristic of early sexual maturity compared with WLS. This study laid a foundation for further identification and analysis of SYF differential metabolites and metabolic pathways using untargeted metabolomics.

A large number of metabolite changes occur during sexual maturity in domestic chickens [50]. In our study, L-arginine, L-prolinamide, (R)-4-hydroxymandelate, homovanillic acid, glutathione, and γ-glutamylcysteine were found using an LC-MS approach. Glutathione and γ-glutamylcysteine are the main antioxidants of animal cells and substrates for GPX4. The entry of cysteine into the cell depends on solute carrier family 7 member 11 *(SLC7A11)* [51], which is considered one of the most critical upstream regulators of ferroptosis. One study showed that a diet supplemented with L-arginine affected gene expression in the mTOR signaling pathway by suppressing the mRNA expression of cathepsin B and the 20S proteasome in the liver [52]. Our findings suggest that L-arginine can improve the oxidative state [53] and improve ovary development during sexual maturity. Homovanillic acid is related to tyrosine metabolism. This result may be related to the melanin deposition and sexual maturation conflict of Wu Liang Shan black-bone chicken. (R)-4-Hydroxymandelate as an intermediated involved in the biosynthesis of the coenzyme Q10 (CoQ10) [54] and plays a key role in mitochondrial oxidative stress [55]. Overall, we speculate that oxidative stress may effect the amino acid metabolism of follicles and lead to early or delayed sexual maturation.

Ferroptosis is a newly discovered type of cell death that mainly depends on iron-mediated oxidative damage and subsequent cell membrane damage [56] and mainly manifests as glutathione (GSH) depletion and glutathione peroxidase 4 (GPX4) inactivation [57,58]. Previous research has shown that the TGF signaling pathway [59], Wnt signaling pathway [60], and cAMP pathway [61] are involved in the regulation of the follicle selection process in domestic chickens. Some studies have focused on ferroptosis induced by oxidative stress in the aquaculture industry and found that heat shock protein *(HSP)*, activating transcription factor 4 *(ATF4)*, solute carrier family 7 member 11 *(SLC7A11),* and *GPX4* were associated with ferroptosis and the mitochondrial pathway [62,63,64]. However, the ferroptosis pathway with respect to poultry reproduction has remained unknown. In our study, we put forward that the ferroptosis pathway may be involved in SYF development for the first time, both for the CH2 and the WLS groups at sexual maturation, thus initiating the biological process of follicle selection. Ferroptosis was found when the most significant differential metabolites were queried in the KEGG database (*p* < 0.05), namely glutathione, oxidized glutathione, and γ-glutamylcysteine. Furthermore, we found that these metabolites were downregulated in the CH2 groups compared with the WLS groups. GSH is an essential cofactor for GPX [65], and its inhibition can induce ferroptosis. Under physiological conditions, system X c- is involved in GSH synthesis. GPX4 and system X c- are important regulatory targets for amino acids and can inhibit ferroptosis. Therefore, we speculated that these differentially expressed metabolites pointed to a novel mechanism of differentially expressed metabolites that take part in protecting the mechanism of CH2 SYF from oxidative stress by suppressing ferroptosis. In the future, we should focus on the important role of ferroptosis in chicken GCs, which may be a novel strategy to study sexual maturation initiation and follicle selection.

The mechanistic target of rapamycin (mTOR) is an evolutionarily conserved serine/threonine protein kinase [66], which functions as a regulator of cell development, metabolism, proliferation, and autophagy [67,68]. mTOR is composed of a complex of mTOR complex 1 *(mTORC1)* and mTOR complex 2 *(mTORC2)* [69,70]. In our study, the mTOR signaling pathway was found when the most significantly differential metabolites were queried in KEGG (*p* < 0.001). Differentially expressed metabolites enriched in the KEGG database, namely AMP-activated protein kinase *(AMP)* and L-Arginine, as well as these differentially expressed metabolites, were significantly downregulated in the CH2 group compared to the WLS group. Previous research indicated that differentially expressed genes *(DEGs)* were associated with high rates of egg production in the chicken hypothalamic–pituitary–ovarian axis via the transcriptome and identified DEGs that regulate the mTOR signaling pathway, the Jak-STAT signaling pathway, tryptophan metabolism, and the PI3K-Akt signaling pathways in the HPO axis in laying hens [71]. In vitro, chicken granulosa cells treated with the mTOR agonist MHY1485 (mTOR activator) showed significantly enhanced granulosa cell proliferation, inhibited apoptosis, and significantly increased mTOR-signaling-related gene (ribosomal protein S6 kinase *(S6K)*, eIF4E binding protein 1 *(4E-BP1)*, and protein kinase C *(PKC))* expression in the peak phase (30-week-old) and late phase (70-week-old) laying hens [72]. Recent evidence also suggests that the mTOR signaling pathway plays important roles in folliculogenesis [73], ovarian cell proliferation, steroidogenesis [74], and oocyte maturation [75]. Under the condition of nutrient deficiency, *AMPK* was a metabolic barrier that could inhibit cell growth and regulate the expression of the proteins *Atg1*, *Atg13,* and *Atg17* via the autophagy signaling pathway by suppressing *mTORC1* activity [76]. *AMPK* and *mTOR* can regulate autophagy through the direct phosphorylation of unc-51-like kinase 1 *(ULK1)* [77]. Therefore, we speculated that these downregulated differentially expressed metabolites were involved in the regulation of sexual maturation through the AMPK-mTOR-autophagy signaling pathway. In our study, we found that mTOR signaling related genes *(SREBP1 and S6K)* were higher in the CH2 group compared to the WLS group. *SERBP1* is a transcription factor and can regulate the expression of enzymes related to lipogenesis and the homeostasis of fat and cholesterol [78]. *S6K* is an extensively studied effector of TORC1. The mTORC1-S6K1 axis controls fundamental cellular processes, including transcription, translation, protein and lipid synthesis, cell growth/size, and cell metabolism [79]. Our study had several limitations, as single omics techniques are often not systematic in interpreting the biological phenomena of complex traits. Based on the current results, future research should confirm these initial findings using multi-omics analyses of important molecular mechanisms that regulate sexual maturation.

## 5. Conclusions

In our study, we revealed potential biomarkers of chicken SYF during sexual maturation using untargeted metabolomics. Fifty-four differentially expressed metabolites were found and were enriched in KEGG. Together, the present findings confirmed that these fifty-four downregulated differentially expressed metabolites were involved in amino acid metabolism and fatty acid metabolism and were related to the state of the body’s antioxidant system. A further novel finding was that the fifty-four differentially expressed metabolites were mainly enriched in the mTOR signaling pathway, the ferroptosis pathway, glutathione metabolism, and ABC transporters. These conclusions have relevant implications, as CH2 regulates self-precocious puberty by downregulating SYF-metabolite-mediated amino acid metabolism related to signaling pathways.

## Figures and Tables

**Table 1:** Dietary composition and nutrient levels.

Items	Amount
**Dietary composition**	
**Corn (%)**	61.0
**Soybean meal (%)**	25.0
**Wheat bran (%)**	3.0
**CaCO_3_ (%)**	8.0
**^a^** **Premix compound (%)**	3.0
**Nutrient levels**	
**ME (MJ/kg^−1^)**	11.30
**CP (%)**	16.0
**Met (%)**	0.38
**Met** + **Cys (%)**	0.62
**Lys (%)**	0.53
**Arg (%)**	0.66
**Thr (%)**	0.46
**Trp (%)**	0.14
**CF (%)**	2.9
**Ash (%)**	16.0
**Ca (%)**	3.50
**TP (%)**	0.60

Abbreviations: ME—metabolizable energy, CP—crude protein, Met—methionine, Cys—cystine, Lys—lysine, Arg—arginine, Thr—threonine, Trp—tryptophan, CF—crude fiber, Ash—crude ash, Ca—calcium, TP—total phosphorus, Cu—Cuprum, Fe—iron, Mn—manganese, Zn—zinc. ^a^ Premix compound included 8000 IU of VA, 5 IU of VE, 0.8 mg of VB_1_, 2.5 mg of VB_2_, 8 mg of Cu, 60 mg of Fe, 60 mg of Mn, and 80 mg of Zn.

**Table 2:** Primer sequences for qPCR.

Gene	GenBank Accession	Primer Sequences (5′→3′)	Size (bp)
***CYP11A1***	NM_001001756.2	AGGGAGAAGTTGGGTGTCTACGA/ CTTGTTGCGGTAGTCACGGTAT	138
***StAR***	NM_204686.3	CCATCTCCTACCAACACCTGC/ CGAGGATGCTGAGTGATTTCTG	240
***CYP19A1***	NM_001001761.4	TGCTGCTCCTGATACTCTGTCC/ AAGTCCACAACTGGCTGGTATCT	201
***FSHR***	NM_205079.2	TTTTCCAGCCTTCCCAAACTAC/ ACCTTATGGACGACGGGTAAAA	152
***SREBP1***	NM_204126.3	AGTGGACCCGTTGGCTCA/ TGAAGGTACTCCAACGCATCC	141
***S6K***	NM_001012587.3	TATGCCTTCCAGACAGATACAAAGC/ GACCAAAGTCTGTCAGCACCAC	238
***GPX4***	NM_001346448.2	AGGGGCTTCGTCTGCATCAT/ TCCTGCTTCCCGAACTGGTT	149
***p53***	NM_205264.1	ATCCTCACCATCCTTACACTGGA/ CCTCATTGATCTCCTTCAGCATCT	280
***SLC7A11***	XM_426289.7	TCCTGCTTTGGGTCTATGAATG/ ACATTATCATTGTGAGAGGGTGCA	163
***β-action***	NM_205518.1	CTGACTGACCGCGTTACTCC/ TTGCACATACCGGAGCCATT	84

Abbreviations: steroidogenic acute regulatory protein (StAR), cytochrome P450 family 11 subfamily A member 1 (CYP11A1), cytochrome P450 family 19 subfamily A member 1 *(CYP19A1)*, glutathione peroxidase 4 (GPX4), solute carrier family 7 member 11 *(SLC7A11)*, ribosomal protein S6 kinase (S6K), sterol regulatory element binding protein 1 (SREBP1), follicle-stimulating hormone receptor *(FSHR)*, tumor protein p53 *(TP53)*.

**Table 3:** Antibody information for immunohistochemistry.

Genes	Primary Ab	Secondary Ab	Dilution Ratio	Resource
***GPX4***	Anti-Rabbit	Peroxidase-Conjugated Goat Anti-Rabbit IgG	1:500/1:200	Wu han servicebio technology CO., LTD
***p53***	Anti-Rabbit	Peroxidase-Conjugated Goat Anti-Rabbit IgG	1:200/1:200	Wu han servicebio technology CO., LTD
***S6K***	Anti-Rabbit	Peroxidase-Conjugated Goat Anti-Rabbit IgG	1:500/1:200	Wu han servicebio technology CO., LTD
***SREBP1***	Anti-Rabbit	Peroxidase-Conjugated Goat Anti-Rabbit IgG	1:1000/1:200	Wu han servicebio technology CO., LTD

**Table 4:** KEGG pathway enrichment analysis of differential metabolites between the CH2 and WLS groups.

Items	Total ^a^	Hits ^b^	*p*-Value	−log10 (*p* Value)	Impact Value ^c^	Metabolites
**mTOR** **signaling pathway**	4	2	0.001	2.7633	0.500	AMP; L-Arginine
**Glutathione metabolism**	38	4	0.003	2.4177	0.3021	Glutathione; Ornithine; Oxidized glutathione; Glutamylcysteine
**Ferroptosis**	29	3	0.013	1.8854	0.2093	Glutathione; Oxidized glutathione; γ-Glutamylcysteine
**ABC transporters**	138	7	0.008	2.0553	0.0507	Glutathione; L-Arginine; Ornithine; Mannitol; Sorbitol; 4-Hydroxyproline; Methyl beta-D-galactosidase
**D-Arginine D-ornithine** **metabolism**	11	2	0.014	1.8349	0.1333	L-Arginine; Ornithine

Abbreviations: ^a^ Total represents the total of metabolites in one pathway. ^b^ Hits represents the number of metabolites in one pathway. ^c^ Impact represents the impact value in one pathway.

## Data Availability

The data that support this study are available in the article.

## References

[1] He H., Li D., Tian Y., Wei Q., Amevor F.K., Sun C., Yu C., Yang C., Du H., Jiang X. (2022). miRNA sequencing analysis of healthy and atretic follicles of chickens revealed that miR-30a-5p inhibits granulosa cell death via targeting Beclin1. J. Anim. Sci. Biotechnol..

[2] Onagbesan O., Bruggeman V., Decuypere E. (2009). Intra-ovarian Growth Factors Regulating Ovarian Function in Avian Species: A Review. J. Anim. Reprod. Sci..

[3] Robinson F.E., Renema R.A., Oosterhoff H.H., Zuidhof M.J., Wilson J.L. (2001). Carcass Traits, Ovarian Morphology and Egg Laying Characteristics in Early Versus Late Maturing Strains of Commercial Egg-Type Hens. Poult. Sci..

[4] Li J., Li C., Li Q., Li G., Li W., Li H., Kang X., Tian Y. (2020). Novel Regulatory Factors in the Hypothalamic-Pituitary-Ovarian Axis of Hens at Four Developmental Stages. Front Genet..

[5] Wang Y., Chen Q., Liu Z., Guo X., Du Y., Yuan Z., Guo M., Kang L., Sun Y., Jiang Y. (2017). Transcriptome Analysis on Single Small Yellow Follicles Reveals That Wnt4 Is Involved in Chicken Follicle Selection. Front Endocrinol..

[6] Lin X. (2018). Degradation and Function of the Regressed Ovarian Follicle in the laying Chickens. Ph.D. Thesis.

[7] Sechman A., Grzegorzewska A.K., Grzesiak M., Kozubek A., Katarzyńska-Banasik D., Kowalik K., Hrabia A. (2020). Nitrophenols suppress steroidogenesis in pre-hierarchical chicken ovarian follicles by targeting STAR, HSD3B1, and CYP19A1 and downregulating LH and estrogen receptor expression. Domest Anim. Endocrinol..

[8] Guo C., Zhang G., Lin X., Zhao D., Zhang C., Mi Y. (2019). Reciprocal stimulating effects of bFGF and FSH on chicken primordial follicle activation through AKT and ERK pathway. Theriogenology.

[9] Chen X., Sun X., Chimbaka I.M., Qin N., Xu X., Liswaniso S., Xu R., Gonzalez J.M. (2021). Transcriptome Analysis of Ovarian Follicles Reveals Potential Pivotal Genes Associated with Increased and Decreased Rates of Chicken Egg Production. Front Genet..

[10] Kim D., Johnson A.L. (2018). Differentiation of the granulosa layer from hen pre-hierarchal follicles associated with follicle-stimulating hormone receptor signaling. Mol. Reprod. Dev..

[11] Nitta H., Osawa Y., Bahr J.M. (1991). Multiple steroidogenic cell populations in the thecal layer of preovulatory follicles of the chicken ovary. Endocrinology.

[12] Cui Z., Amevor F.K., Feng Q., Kang X., Song W., Zhu Q., Wang Y., Li D.Y., Zhao X.L. (2020). Sexual Maturity Promotes Yolk Precursor Synthesis and Follicle Development in Hens via Liver-Blood-Ovary Signal Axis. Animals.

[13] Hrabia A., Sechman A., Gertler A., Rząsa J. (2011). Effect of Growth Hormone on Steroid Content, Proliferation and Apoptosis in the Chicken Ovary during Sexual Maturation. J. Cell Tissue Res..

[14] Johnson A.L. (2015). Ovarian follicle selection and granulosa cell differentiation. Poult Sci..

[15] Kang L., Zhang Y., Zhang N., Zang L., Wang M., Cui X., Jiang Y. (2012). Identification of differentially expressed genes in ovaries of chicken attaining sexual maturity at different ages. Mol. Biol. Rep..

[16] Li J., Luo W., Huang T., Gong Y. (2019). Growth differentiation factor 9 promotes follicle-stimulating hormone-induced progesterone production in chicken follicular granulosa cells. Gen. Comp. Endocrinol..

[17] Tang S., Li X., Wu X., Gong Y. (2022). WT1 suppresses follicle-stimulating hormone-induced progesterone secretion by regulating ERK1/2 pathway in chicken preovulatory granulosa cells. Genes.

[18] Qin N., Tyasi T.L., Sun X., Chen X., Zhu H., Zhao J., Xu R. (2020). Determination of the roles of GREM1 gene in granulosa cell proliferation and steroidogenesis of hen ovarian prehierarchical follicles. Theriogenology.

[19] Du H., Guo Y., Wu X., Gong Y. (2022). FOXL2 regulates the expression of the COL4A1 collagen gene in chicken granulosa cells. Mol. Reprod. Dev..

[20] Ahumada-Solórzano S.M., Martínez-Moreno C.G., Carranza M., Ávila-Mendoza J., Luna-Acosta J.L., Harvey S., Luna M., Arámburo C. (2016). Autocrine/paracrine proliferative effect of ovarian GH and IGF-I in chicken granulosa cell cultures. Gen. Comp. Endocrinol..

[21] Lin J.X., Jia Y.D., Zhang C.Q. (2011). Effect of epidermal growth factor on follicle-stimulating hormone-induced proliferation of granulosa cells from chicken pre-hierarchical follicles. J. Zhejiang Univ. Sci..

[22] Kim D., Johnson A.L. (2016). Vasoactive intestinal peptide promotes differentiation and clock gene expression in granulosa cells from pre-hierarchal follicles. Mol. Reprod. Dev..

[23] Wei Q., Li J., He H., Cao Y., Li D., Amevor F.K., Zhang Y., Wang J., Yu C., Yang C. (2022). miR-23b-3p inhibits chicken granulosa cell proliferation and steroid hormone synthesis via targeting GDF9. Theriogenology.

[24] Wu X., Zhang N., Li J., Zhang Z., Guo Y., Li D., Zhang Y., Gong Y., Jiang R., Li H. (2022). gga-miR-449b-5p Regulates Steroid Hormone Synthesis in Laying Hen Ovarian Granulosa Cells by Targeting the IGF2BP3 Gene. Animals.

[25] Zhou Y., Liu J., Lei Q., Han H., Liu W., Cun W.T., Li F., Cao D. (2020). Transcriptome Analysis of the Chicken Follicular Theca Cells with miR-135a-5p Suppressed. G3 Genes Genomes Genet..

[26] Johnson C.H., Ivanisevic J., Siuzdak G. (2016). Metabolomics: Beyond biomarkersand towards mechanisms. J. Nat. Rev. Mol. Cell Biol..

[27] Cui L., Lu H., Lee Y.H. (2018). Challenges and emergent solutions for LC-MS/MS based untargeted metabolomics in diseases. Mass Spectrum. Rev..

[28] Bujak R., Struck-Lewicka W., Markuszewski M.J., Kaliszan R. (2015). Metabolomics for laboratory diagnostics. J. Pharm. Biomed. Anal..

[29] Fiehn O. (2022). Metabolmics-the link between genotypes and phenotypes. Plant Mol. Bio..

[30] Zelena E., Dunn W.B., Broadhurst D., Francis-McIntyre S., Carroll K.M., Begley P., O’Hagan S., Knowles J.D., Halsall A. (2009). Development of a Robust and Repeatable UPLC-MS Method for the Long-Term Metabolomic Study of Human Serum. J. Anal. Chem..

[31] Want E.J., Masson P., Michopoulos F., Wilson I.D., Theodoridis G., Plumb R.S., Shockcor J., Loftus N., Holmes E., Nicholson J.K. (2013). Global metabolic profiling of animal and human tissues via UPLC-MS. Nat. Protoc..

[32] Wishart D.S., Dan T., Knox C., Eisner R., Guo A.C., Young N., Fung C., Block D., Lewis M., Guo N. (2007). HMDB: The human metabolome database. J. Nucleic Acids Res..

[33] Horai H., Arita M., Kanaya S., Nihei Y., Ikeda T., Suwa K., Ojima Y., Tanaka K., Tanaka S., Aoshima K. (2010). MassBank: A public repository for sharing mass spectral data for life sciences. Int. J. Mass Spectr..

[34] Sud M., Fahy E., Cotter D., Brown A., Dennis E.A., Glass C.K., Merrill A.H., Murphy R.C., Raetz C.R., Russell D.W. (2007). LMSD: LIPID MAPS structure database. J. Nucleic Acids Res..

[35] Abdelrazig S., Safo L., Rance G.A., Fay M.W., Theodosiou E., Topham P.D., Kim D.H., Fernández-Castané A. (2020). Metabolic characterisation of Magnetospirillum gryphiswaldense MSR-1 using LC-MS-based metabolite profiling. J. RSC Adv..

[36] Ogata H., Goto S., Sato K., Fujibuchi W., Bono H., Kanehisa M. (1999). KEGG: Kyoto Encyclopedia of Genes and Genomes. J. Nucleic Acids Res..

[37] Thévenot E.A., Roux A., Xu Y., Ezan E., Junot C. (2015). Analysis of the human adult urinary metabolome variations with age, body mass index, and gender by implementing a comprehensive workflow for univariate and OPLS statistical Analyses. J. Proteome Res..

[38] Xia J., Wishart D.S. (2011). Web-based inference of biological patterns, functions and pathways frommetabolomic data using MetaboAnalyst. Nat. Protocol..

[39] Miao Z., Miao Z., Teng X., Xu S. (2022). Melatonin alleviates lead-induced intestinal epithelial cell pyroptosis in the common carps (*Cyprinus carpio*) via miR-17-5p/TXNIP axis. Fish Shellfish. Immunol..

[40] Sun Q., Liu Y., Teng X., Luan P., Teng X., Yin X. (2022). Immunosuppression participated in complement activation-mediated inflammatory injury caused by 4-octylphenol via TLR7/IκBα/NF-κB pathway in common carp (*Cyprinus carpio)* gills. Aquat. Toxicol..

[41] Mabuchi R., Adachi M., Ishimaru A., Zhao H., Tanimoto S. (2019). Changes in Metabolic Profiles of Yellow tail (Seriola Quinqueradiata) Muscle during Cold Storage as a Freshness Evaluation Tool Based on GC-MS Metabolomics. Foods.

[42] Wang Y., Yuan J., Sun Y., Li Y., Wang P., Shi L., Ni A., Zong Y., Zhao J., Bian S. (2022). Genetic Basis of Sexual Maturation Heterosis: Insights From Ovary lncRNA and mRNA Repertoire in Chicken. Front Endocrinol..

[43] Sharp P.J. (1993). Photoperiodic Control of Reproduction in the Domestic Hen1. Poult. Sci..

[44] Shirtcliff E.A., Dahl R.E., Pollak S.D. (2009). Pubertal development: Correspondence between hormonal and physical development. Child Dev..

[45] Johnson A.L., Solovieva E.V., Bridgham J.T. (2002). Relationship between steroidogenic acute regulatory protein expression and progesterone production in hen granulosa cells during follicle development. Biol. Reprod..

[46] Zhou S., Ma Y., Zhao D., Mi Y., Zhang C. (2020). Transcriptome profiling analysis of underlying regulation of growing follicle development in the chicken. Poult Sci..

[47] Zhang T., Chen L., Han K., Zhang X., Zhang G., Dai G., Wang J., Xie K. (2019). Transcriptome analysis of ovary in relatively greater and lesser egg producing Jinghai Yellow Chicken. Anim. Reprod. Sci..

[48] Lew P.D., Tyler N.C., Gous R.M., Dunn I.C., Sharp P.J. (2008). Photoperiodic response curves for plasma LH concentrations and age at first egg in female broiler breeders. Anim. Reprod. Sci..

[49] Dunn I.C., Lewis P.D., Wilson P.W., Sharp P.J. (2003). Acceleration of maturation of FSH and LH responses to photostimulation in prepubertal domestic domestic hens by–oestrogen. Reproduction.

[50] Cui Z., Ning Z., Deng X., Du X., Amevor F.K., Liu L., Kang X., Tian Y., Wang Y., Li D. (2022). Integrated Proteomic and Metabolomic Analyses of Chicken Ovary Revealed the Crucial Role of Lipoprotein Lipase on Lipid Metabolism and Steroidogenesis During Sexual Maturity. Front. Physiol..

[51] Zheng J., Conrad M. (2020). The Metabolic Underpinnings of Ferroptosis. Cell. Metab..

[52] Yuan C., Bu X.C., Yan H.X., Lu J.J., Zou X.T. (2016). Dietary L-arginine levels affect the liver protein turnover and alter the expression of genes related to protein synthesis and proteolysis of laying hens. Poult. Sci..

[53] Ragy M.M., Abdel-Hamid H.A., Toni N.D.M. (2019). Pathophysiological changes in experimental polycystic ovary syndrome in female albino rats: Using either hemin or L-arginine. J. Cell Physiol..

[54] Banh R.S., Kim E.S., Spillier Q., Biancur D.E., Yamamoto K., Sohn A.S.W., Shi G., Jones D., Kimmelman A.C., Pacold M.E. (2021). The polar oxy-metabolome reveals the 4-hydroxymandelate CoQ10 synthesis pathway. Nature.

[55] Hargreaves I., Heaton R.A., Mantle D. (2020). Disorders of Human Coenzyme Q10 Metabolism: An Overview. Int. J. Mol. Sci..

[56] Chen X., Kang R., Kroemer G., Tang D. (2021). Broadening horizons: The role of ferroptosis in cancer. Nat. Rev. Clin. Oncol..

[57] Wu J., Wang Y., Jiang R., Xue R., Yin X., Wu M., Meng Q. (2021). Ferroptosis in liver disease: New insights into disease mechanisms. Cell Death Discov..

[58] Seibt T.M., Proneth B., Conrad M. (2019). Role of GPX4 in ferroptosis and its pharmacological implication. J. Free Radic. Biol. Med..

[59] Nie R., Zheng X., Zhang W., Zhang B., Ling Y., Zhang H., Wu C. (2022). Morphological Characteristics and Transcriptome Landscapes of Chicken Follicles during Selective Development. Animals.

[60] Zhong C., Wang Y., Liu C., Jiang Y., Kang L. (2022). A Novel Single-Nucleotide Polymorphism in WNT4 Promoter Affects Its Transcription and Response to FSH in Chicken Follicles. Genes.

[61] Wang J., Zhao C., Li J., Feng Y., Gong Y. (2017). Transcriptome analysis of the potential roles of FOXL2 in chicken pre-hierarchical and pre-ovulatory granulosa cells. Genomics.

[62] Cui J., Zhou Q., Yu M., Liu Y., Teng X.H., Gu X.H. (2022). 4-tert-butylphenol triggers common carp hepatocytes ferroptosis via oxidative stress, iron overload, SLC7A11/GSH/GPX4 axis, and ATF4/HSPA5/GPX4 axis. Ecotoxicol. Environ. Saf..

[63] Zhang J., Cui J., Wang Y., Lin X., Teng X.H., Tang Y. (2022). Complex molecular mechanism of ammonia-induced apoptosis in chicken peripheral blood lymphocytes: miR-27b-3p, heat shock proteins, immunosuppression, death receptor pathway, and mitochondrial pathway. Ecotoxicol Environ. Saf..

[64] Chen D., Liang J., Jiang C., Wu D., Huang B., Teng X., Tang Y. (2022). Mitochondrion Participated in Effect Mechanism of Manganese Poisoning on Heat Shock Protein and Ultrastructure of Testes in Chickens. Biol Trace Elem. Res..

[65] Forcina G.C., Dixon S.J. (2019). GPX4 at the Crossroads of Lipid Homeostasis and Ferroptosis. J. Proteom..

[66] Zhou X., Zhang H., Zong N. (2018). Effects of rapamycin and mammalian target of rapamycin (mTOR) on immune regulation advance. J. Chin J. Cell. Mol. Immunol..

[67] Couso I., Evans S.B., Li J., Liu Y., Ma F.F., Diamond S., Allen D.K., Umen J.G. (2016). Synergism between inositol polyphosphates and TOR kinase signaling in nutrient sensing, growth control, and lipid metabolism in Chlamydomonas. The Plant Cell..

[68] Guo Z., Yu Q. (2019). Role of mTOR Signaling in Female Reproduction. Front Endocrinol..

[69] Condon K.J., Sabatini D.M. (2019). Nutrient regulation of mTORC1 at a glance. J. Cell Sci..

[70] Fu W., Hall M.N. (2020). Regulation of mTORC2 Signaling. Genes.

[71] Mishra S.K., Chen B., Zhu Q., Xu Z., Ning C., Yin H., Wang Y., Zhao X., Fan X., Yang M. (2020). Transcriptome analysis reveals differentially expressed genes associated with high rates of egg production in chicken hypothalamic-pituitary-ovarian axis. Sci. Rep..

[72] Hao E.Y., Wang D.H., Chen Y.F., Zhou R.Y., Chen H., Huang R.L. (2021). The relationship between the mTOR signaling pathway and ovarian aging in peak-phase and late-phase laying hens. Poult Sci..

[73] Herta A.C., Lolicato F., Smitz J.E.J. (2018). In vitro follicle culture in the context of IVF. Reproduction.

[74] Palaniappan M., Menon K.M. (2012). Luteinizing hormone/human chorionic gonadotropin-mediated activation of mTORC1 signaling is required for androgen synthesis by theca-interstitial cells. J. Mol. Endocrinol..

[75] Guo J., Zhang T., Guo Y.S., Sun T., Li H., Zhang X.Y., Yin H., Cao G.Y., Yin Y.X., Wang H. (2018). Oocytestage-specific effects of mTOR determine granulosa cell fate andoocyte quality in mice. Proc. Natl. Acad. Sci. USA.

[76] Mizushima N., Levine B., Cuervo A., Klion D. (2008). Autophagy flights disease through cellular self-digestion. Nature.

[77] Kim J., Kundu M., Viollet B., Guan K.L. (2011). AMPK and mTOR regulate autophagy through direct phosphorylation of Ulk1. Nature Cell Bio..

[78] Yan R., Cao P., Song W., Li Y., Wang T., Qian H., Yan C., Yan N. (2021). Structural basis for sterol sensing by Scap and Insig. Cell Rep..

[79] Magnuson B., Ekim B., Fingar D.C. (2012). Regulation and function of ribosomal protein S6 kinase (S6K) within mTOR signaling networks. Biochem. J..

